# Stochastic Resonance in an Underdamped System with Pinning Potential for Weak Signal Detection

**DOI:** 10.3390/s150921169

**Published:** 2015-08-28

**Authors:** Haibin Zhang, Qingbo He, Fanrang Kong

**Affiliations:** Department of Precision Machinery and Precision Instrumentation, University of Science and Technology of China, Hefei 230026, China; E-Mails: zhbzhbyr@mail.ustc.edu.cn (H.Z.); kongfr@ustc.edu.cn (F.K.)

**Keywords:** underdamped stochastic resonance, potential model, nonlinear filter, weak signal detection, fault diagnosis

## Abstract

Stochastic resonance (SR) has been proved to be an effective approach for weak sensor signal detection. This study presents a new weak signal detection method based on a SR in an underdamped system, which consists of a pinning potential model. The model was firstly discovered from magnetic domain wall (DW) in ferromagnetic strips. We analyze the principle of the proposed underdamped pinning SR (UPSR) system, the detailed numerical simulation and system performance. We also propose the strategy of selecting the proper damping factor and other system parameters to match a weak signal, input noise and to generate the highest output signal-to-noise ratio (SNR). Finally, we have verified its effectiveness with both simulated and experimental input signals. Results indicate that the UPSR performs better in weak signal detection than the conventional SR (CSR) with merits of higher output SNR, better anti-noise and frequency response capability. Besides, the system can be designed accurately and efficiently owing to the sensibility of parameters and potential diversity. The features also weaken the limitation of small parameters on SR system.

## 1. Introduction

Weak signal detection is a challenging task in sensor signal detection and early machinery fault diagnosis. Conventional detection methods are limited by strong noise conditions. Hence, noise usually has a disruptive role in nature. However, there is a situation, in which noise helps to make the system behave in a more coherent manner. This intriguing and rather counterintuitive phenomenon is called stochastic resonance (SR) [1]. Since proposed by Benzi *et al.* in 1981 as an explanation of the observed periodicity in the ice ages on earth, SR has become an attractive research topic in the field of nonlinear science [2]. During the past decades, SR has been demonstrated experimentally in a large variety of physical, biological, electrical, optical systems [3,4,5] and widely adopted in amplification of weak signals in different research fields.

Jung firstly investigated the cooperative effect of noise and periodic driving in bi-stable system as well as the application of SR system for the detection and amplification of weak signal with heavy background noise [3]. The optimal nonlinear detection with SR by a threshold device of a periodic train of soliton-like pulses embedded in arbitrarily distributed white noise was studied in [6]. Twice sampling SR technique for detection of weak signal under large parameters in terms of the theory of adiabatic elimination was proposed by Leng and Wang [7]. The problem of cascaded bi-stable stochastic resonance systems with large parameters was addressed and nonlinear low-pass filter characteristics were revealed in [8]. A new method of multi-scale noise tuning was also developed to realize the SR at a fixed noise level by transforming the noise at multiple-scales to be distributed in an approximate 1/*f* form [9,10]. Adaptive and fast SR method based on dyadic wavelet transform and least square system parameters solving was proposed by Qin and his co-workers [11]. Detecting faint signals with strong noises in sensors by modified adaptive SR was presented in [12]. Besides, SR systems with many new potential models were also proposed by researchers like the tri-stable potential [13,14,15], multi-stable SR [16], joint Woods-Saxon and Gaussian potential [17], *etc.* These studies indicate that SR exists in different systems consisting of potential model, weak driving signal and noise, besides the weak signals can be enhanced with the assistance of proper intensity of noise.

It should be noted that most of the signal detection methods via SR systems are based on the first-order over-damped SR models, *i.e.*, the system inertia is ignored when compared to the damping factor and it is normalized for simplicity. In fact, both the system inertia and the damping factor have effects on SR realization and make a difference on the system output. Thus the damping factor has already been considered by many researchers [18,19,20,21,22,23] as it is beneficial to obtain output signal of higher signal-to-noise ratio (SNR). For periodically driven underdamped bi-stable systems, an intra-well stochastic resonance can exist, together with the conventional inter-well SR as shown in [18]. The method of moments was applied to an underdamped bi-stable oscillator for observations of SR by Kang [19], where the system was driven by Gaussian white noise and a weak periodic force. A detailed numerical investigation of SR in underdamped systems was then carried out by Ray [20]. Signal amplification via a nano-mechanical Duffing resonator SR was experimentally studied in literature [21]. SR in an underdamped bi-stable system subjected to a weak asymmetric dichotomous noise was explored numerically by Xu [22]. Reference [23] presented a new weak signal detection method based on the van der Pol-Duffing oscillator.

Considering the aforementioned studies, a deeper discussion of the underdamped SR system with a novel pinning potential model is presented. The model was firstly proposed for magnetic domain walls (DWs) in ferromagnetic strips. In the recent studies, SR phenomenon of a single DW in a ferromagnetic stripe with two pinning sites was observed. Under a weak oscillating field, the wall performs irregular transitions between both constrictions in the presence of thermal fluctuations and the results indicate that synchronized wall transitions with the driving field can be achieved at the optimal level of noise [24,25]. Inspired by the novel SR phenomenon, we extend the new second order system of an underdamped pinning SR (UPSR) to the area of weak signal detection. In the potential model, several new parameters are considered, which affect the potential shape independently and also it is always finite when compared to the polynomial potential model. This is conducive to the parameters optimization procedure as well as the final output. Then, combining the UPSR with a parameter tuning method, we propose a new strategy to realize weak signal detection for bearing fault diagnosis. Some significant superiorities are revealed by the latter studies, which means that better anti-noise ability and frequency response are found in detection of signal with strong background noise. It possesses better features of the nonlinear filtering via the pinning potential and the system can be improved more accurately and easily due to its parameter sensibility.

The following parts of the paper are arranged as follows. The theory of UPSR and the numerical analysis method are introduced in Section 2, where the novel potential model is analyzed and a weak signal detection scheme is discussed. Section 3 shows the numerical analysis employing simulation signals to evaluate the UPSR performance. Section 4 verifies the practicability of the proposed method referring to sets of defective bearing signals and presents further discussions and research prospects. Finally we draw some conclusions in Section 5.

## 2. Underdamped Pinning Stochastic Resonance

### 2.1. Pinning Potential Model in Ferromagnetic Strips

According to the existing research, the basis of classical SR phenomenon can be described as in [1]: a particle is driven by a periodic signal and the random noise in a bi-stable potential which consists of two potential wells and one potential barrier. Then the periodic oscillation can be enhanced by the assistance of proper noise. SR has been theoretically developed in traditional bi-stable systems. When the inertia is ignored, in which case the system is over-damped one, we have the dynamical equation of a CSR system as denoted by the first equation of Equation (1). Considering both the inertia and the damping factor of the system, the expression of its Langevine equation is denoted as the second one of the equations shown as Equation (1).

(1) $$\{\begin{array}{c} \;\frac{dx}{dt}=-V^{\prime }\left(x\right)+s\left(t\right)+n\left(t\right)\;\\ \frac{d^{2}x}{dt^{2}}=-V^{\prime }\left(x\right)-\gamma \frac{dx}{dt}+s\left(t\right)+n\left(t\right) \end{array}$$

For the CSR system, the potential V(x) with polynomial form is:

(2) $$V\left(x\right)=-\frac{a}{2}x^{2}+\frac{b}{4}x^{4}$$

Which is a typical bi-stable potential function representing a reflection-symmetric quartic potential. *a* and *b* are real parameters of system. In Equation (1), $n\left(t\right)=\sqrt{2D}\xi \left(t\right)$ represents the zero-mean Gaussian white noise with n(t)n(t+τ)=2Dδ(t) where $\sqrt{2D}$ is the noise intensity. $s\left(t\right)=Acos\left(2\text{π}f_{d}t+\text{φ}\right)$ means the original periodic driving signal with amplitude of A. *f_d_* indicates the driving frequency and γ in Equation (1) represents the damping factor. In order to take the new pinning potential model [24,25] into consideration, the potential profile is described by the following function:

(3) $$V_{pin}\left(x\right)=V_{0}-V_{d}\left(\text{exp}\left(-\frac{{\left(x+x_{1}\right)}^{2}}{L^{2}}\right)+\text{exp}\left(-\frac{{\left(x+x_{2}\right)}^{2}}{L^{2}}\right)\right)$$

In Equation (3), *V_0_* is a constant, *x_1_* and *x_2_* are the centers of each pinning site, *L* and *V_d_* are the length and the effective depth of each pinning site [24,25], which are positive real numbers. In this paper, they act as the system parameters together. For a symmetric bi- or mono- stable potential model, *x_1_* and *x_2_* are replaced by $\pm x_{0}$ and the constant *V_0_* is ignored as it is good for nothing in the SR model. Apparently, the parameter *V_d_* acts on the depth of potential well and *x_0_* mainly influences the well location. *L* is reflection of the height of potential barrier, which actually depends on the magnitude relation between *L* and $\left|x_{0}\right|$. It also affects the potential shape, which means a bi-stable or mono-stable one. Hence, the potential shape can be adjusted conveniently by tuning parameters *V_d_*, *x_0_* and *L* separately or jointly.

The equilibrium points of the system in Equation (3) stands at the positions where the pinning force equals to 0:

(4) $$F_{pin}\left(x\right)=V_{pin}^{\prime }\left(x\right)=V_{d}\left(\frac{2\left(x+x_{0}\right)}{L^{2}}\times \exp\left(-\frac{{\left(x+x_{0}\right)}^{2}}{L^{2}}\right)+\frac{2\left(x-x_{0}\right)}{L^{2}}\times \exp\left(-\frac{{\left(x-x_{0}\right)}^{2}}{L^{2}}\right)\right)=0$$

**Figure 1:**
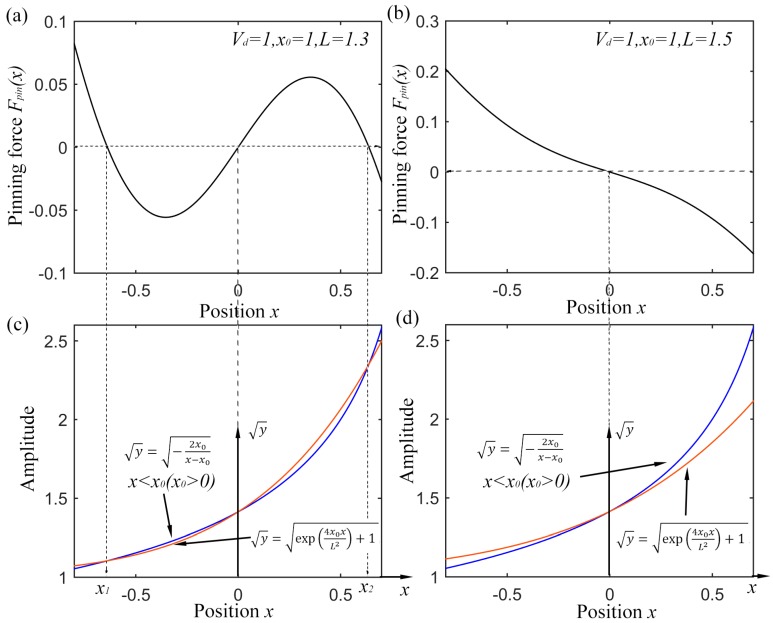
Distribution of pinning force $F_{pin}\left(x\right)$ with different position *x*, (**a**) pinning force distribution of bi-stable state; (**b**) pinning force distribution of mono-stable state; (**c**) curves of Equation (5) for bi-stable state; (**d**) curves of Equation (5) for mono-stable state.

The distribution of pinning force $F_{pin}\left(x\right)$ with different position *x* is depicted in Figure 1a,b with different parameters. Simplifying Equation (4), there is the expression:

(5) $$g_{1}\left(x\right)\equiv -\frac{2x_{0}}{x-x_{0}}=g_{2}\left(x\right)\equiv \exp\left(\frac{4x_{0}x}{L^{2}}\right)+1$$

The first two figures in Figure 1 stand for two different states of potential model. *L* = 1.3 indicates a bi-stable one and *L* = 1.5 results in a mono-stable one. In order to reveal the equilibrium position, the curves of both the right and left side of Equation (5) with extraction operation for a high contrast ratio are shown in Figure 1c,d. For a certain value of *x_0_*, $\sqrt{y}=\sqrt{g_{1}\left(x\right)}=\sqrt{-\frac{2x_{0}}{x-x_{0}}}$ (*x < x_0_*) has the decided form as the blue lines with an asymptotic line of $\sqrt{y}=\sqrt{x_{0}}$. The red lines indicate the distribution of $\sqrt{y}=\sqrt{g_{2}\left(x\right)}=\sqrt{\exp\left(\frac{4x_{0}x}{L^{2}}\right)+1\;}$. If *L* equals 1.5 in Figure 1d, the slope of $g_{2}\left(x\right)$ at *x* = 0 is smaller than that of $g_{1}\left(x\right)$, which results in only one equilibrium point and a mono-stable potential model. While it is the different case as Figure 1c for *L* = 1.3 which brings a larger slope of $g_{2}\left(x\right)$ at *x* = 0. This makes the two lines have another intersection among $x\in \left(0,x_{0}\right)$. It is also easy to demonstrate that if $g_{1}\left(x_{1}\right)=g_{2}\left(x_{1}\right)$, then $g_{1}\left(-x_{1}\right)=g_{2}\left(-x_{1}\right)$. So there exists the third intersection among $x\in \left(-x_{0},0\right)$. Under the circumstance, the potential appears to be bi-stable. The critical condition for the two different state are $g_{1}\prime \left(0\right)=g_{2}\prime \left(0\right)$ with a final result of:

(6) $$L=\sqrt{2}x_{0}$$

According to the above analysis, the pinning potential of Equation (3) presents a bi-stable model with $0<L<\sqrt{2}x_{0}$, while a mono-stable one with $L\geq \sqrt{2}x_{0}$. The equilibrium points locate among (−*x_0_*, *x_0_*). Hence the potential shape can be adjusted by tuning parameters $V_{d}$, $x_{0}$ and *L* separately. This makes the system different from the CSR system which is only a bi-stable one. The two states can be reached just by tuning the system parameters, so the UPSR system has potential diversity, which benefits the acquisition of better output.

### 2.2. Underdamped Pinning SR and Numerical Solution

Substituting the reduced form of $V_{pin}^{\prime }\left(x\right)$ in Equation (4) into second formula of Equation (1), the governing equation of SR based on the pinning potential is denoted as:

(7) $$\frac{d^{2}x}{dt^{2}}=-V_{d}\left(\frac{2\left(x+x_{0}\right)}{L^{2}}\times \exp\left(-\frac{{\left(x+x_{0}\right)}^{2}}{L^{2}}\right)+\frac{2\left(x-x_{0}\right)}{L^{2}}\times \exp\left(-\frac{{\left(x-x_{0}\right)}^{2}}{L^{2}}\right)\right)-\gamma \frac{dx}{dt}+\left(s\left(t\right)+n\left(t\right)\right)$$

**Figure 2:**
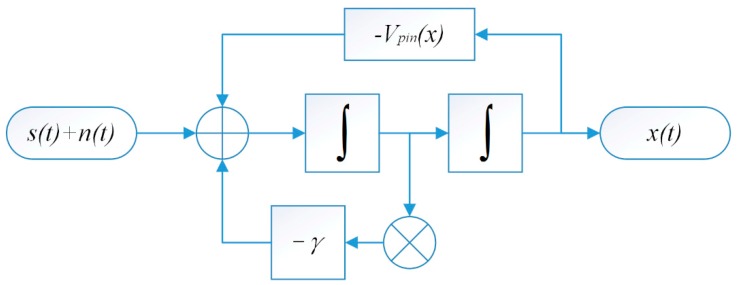
System model of UPSR.

The SR system of Equation (7) is illustrated in system chart of Figure 2. A secondary integration process is applied in fact for an underdamped SR system and the calculation of output *x(t)* means a secondary filtering process for the input signal. According to Equation (7), the system output *x(t)* (left side of Equation (7)) is determined by the potential (first item of right hand side of Equation (4)), damping item (middle item) and the input signal (third item). Then the system output can be adjusted by tuning the parameters $V_{d}$, $x_{0}$, *L* and γ for the optimal output. Note that when the damping factor γ is large enough and the equation is normalized, inertia term on the left side can be omitted, which performs as an approximate over-damped SR. In other words, the SR system with role of damping factor possesses better adaptability to input signals with different noise intensity by tuning the damping factor and system parameters than the traditional over-damped SR.

Subsequently, in order to solve the second-order differential equation, Equation (7) is separated into two first-order differential equations by assuming $\frac{dx}{dt}=y$ as:

(8) $$\{\begin{array}{c} \frac{dx}{dt}=y\;\\ \frac{dy}{dt}=-V_{pin}^{\prime }\left(x\right)-\gamma y+\left(s\left(t\right)+n\left(t\right)\right) \end{array}$$

By linearizing Equation (7) with *D* = 0 and *A* = 0 at the three singular points P_s1_(*x_s_*, 0), P_s2_(*x_-s_*, 0) and P_u_(0,0), we have the eigenvalues of the characteristic equation:

(9) $$\{\begin{array}{c} \beta_{1,2}=\frac{-\gamma \pm \sqrt{\gamma^{2}-4\alpha }}{2}\\ \lambda_{1,2}=\frac{-\gamma \pm \sqrt{\gamma^{2}-4\alpha_{0}}}{2} \end{array}$$

In the above formula, α and α*0* stand for the linearization parameters of the $V_{pin}^{\prime }\left(x\right)$:

(10) $$\{\begin{array}{c} \alpha =V_{d}\frac{4x_{0}\left(2x_{0}^{2}-2x_{\pm s}^{2}-L^{2}\right)}{L^{4}\left(x_{\pm s}-x_{0}\right)}exp\left(-\frac{{\left(x_{\pm s}-x_{0}\right)}^{2}}{L^{2}}\right)\\ \alpha_{0}=-V_{d}\frac{4\left(2x_{0}^{2}-L^{2}\right)}{L^{4}}exp\left(-\frac{{x_{0}}^{2}}{L^{2}}\right) \end{array}$$

According to the Kramers Function, assuming ρ(*x, y, t*) to signify the probability density of system (8) at time *t* and $f\left(x\right)=-V_{pin}^{\prime }\left(x\right)+Acos\left(2\text{π}f_{d}t\right)$, the corresponding Fokker-Plank equation (FPE) is denoted as:

(11) $$\frac{\partial \text{ρ}\left(x,\;y,\;t\right)}{\partial t}=-\frac{\partial }{\partial x}\left(y\text{ρ}\left(x,\;y,\;t\right)\right)-\frac{\partial }{\partial y}\left(\left(-\gamma y+f\left(x\right)\right)\text{ρ}\left(x,\;y,\;t\right)\right)+D\frac{\partial^{2}}{\partial y^{2}}\text{ρ}\left(x,\;y,\;t\right)$$

Then the quasi-steady-state distribution function of system (8) is expressed as:

(12) $$\rho_{st}\left(x,\;y,\;t\right)=Nexp\left(-\frac{\tilde{U}\left(x,y,t\right)}{D}\right)$$

In Equation (12), *N* represents the normalization constant, and $\tilde{U}\left(x,y,t\right)$ is generalized potential function with the form as Equation (13) by utilizing the small parameters expansions:

(13) $$\tilde{U}\left(x,y,t\right)=\frac{\gamma }{2}y^{2}+\text{γ}\int^{\text{​}}-f\left(x\right)dx=\frac{\gamma }{2}y^{2}+V_{pin}\left(x\right)-Axcos\left(2\text{π}f_{d}t\right)$$

Via the probability transition rate for Brownian particle motion is a two-dimensional bi-table system, the transition rate between the two wells denotes:

(14) $$R_{\pm }\left(t\right)=\frac{\sqrt{{\text{β}}_{1}{\text{β}}_{2}}}{2\text{π}}\sqrt{-\frac{{\text{λ}}_{1}}{{\text{λ}}_{2}}}\exp\frac{\gamma }{D}\left(2V_{d}\left(\frac{x_{0}}{x_{\pm s}-x_{0}}\exp\left(-\frac{{\left(x_{\pm s}+x_{0}\right)}^{2}}{L^{2}}\right)-\exp\left(-\frac{{x_{0}}^{2}}{L^{2}}\right)\right)-Ax_{\pm s}cos\left(2\text{π}f_{d}t\right)\right)$$

Equation (14) can be transformed into the form of a Taylor series:

(15) $$R_{\pm }\left(t\right)=R_{0}\left(1\mp Ax_{\pm s}cos\left(2\text{π}f_{d}t\right)+\frac{1}{2}{\left(Ax_{\pm s}\right)}^{2}cos^{2}\left(2\text{π}f_{d}t\right)\pm \ldots \right)$$

where

(16) $$R_{0}=\frac{\sqrt{{\text{β}}_{1}{\text{β}}_{2}}}{2\text{π}}\sqrt{-\frac{{\text{λ}}_{1}}{{\text{λ}}_{2}}}\exp\frac{\gamma }{D}\left(2V_{d}\left(\frac{x_{0}}{\left|x_{\pm s}\right|-x_{0}}\exp\left(-\frac{{\left(\left|x_{\pm s}\right|+x_{0}\right)}^{2}}{L^{2}}\right)-\exp\left(-\frac{{x_{0}}^{2}}{L^{2}}\right)\right)\right)$$

Supposing

(17) $$R_{1}\text{β}=R_{0}\frac{\gamma }{D}\left|x_{\pm s}\right|A$$

The output spectrum of system (7) is expressed in the form:

(18) $$S\left(\text{ω}\right)=S_{1}\left(\text{ω}\right)+S_{2}\left(\text{ω}\right)$$

In the power spectrum of system output, *S_1_(*ω*)* stands for the power spectrum of periodic driving signal and *S_2_(*ω*)* means the one of noise. With the results in Equations (16) and (17), we have:

(19) $$\{\begin{array}{c} S_{1}\left(\text{ω}\right)=\frac{\text{π}{x_{\pm s}}^{2}{\left(R_{1}\text{β}\right)}^{2}}{2\left(R_{0}^{2}+{\text{ω}}_{d}^{2}\right)}\left[\delta \left(\text{ω}-{\text{ω}}_{d}\right)+\delta \left(\text{ω}-{\text{ω}}_{d}\right)\right]\\ S_{2}\left(\text{ω}\right)=\left[1-\frac{{\left(R_{1}\text{β}\right)}^{2}}{2\left(R_{0}^{2}+{\text{ω}}_{d}^{2}\right)}\right]\cdot \frac{2{x_{\pm s}}^{2}R_{0}}{R_{0}^{2}+{\text{ω}}^{2}} \end{array}$$

In Equation (19), ${\text{ω}}_{d}=2\text{π}f_{d}$ means the driving angular frequency of the input signal. The results also indicate that the *S_1_(*ω*)* comes from the input periodic signal with *S_2_(*ω*)* deriving from the input noise. Considering the positive component of driving frequency, the output SNR is further defined as:

(20) $$SNR=\frac{\int_{0}^{\infty }S_{1}\left(\text{ω}\right)d\text{ω}}{S_{2}\left(\text{ω}={\text{ω}}_{d}\right)}=\frac{\text{π}{\left(R_{1}\text{β}\right)}^{2}}{4R_{0}\left[1-\frac{{\left(R_{1}\text{β}\right)}^{2}}{2\left(R_{0}^{2}+{\text{ω}}_{d}^{2}\right)}\right]}$$

**Figure 3:**
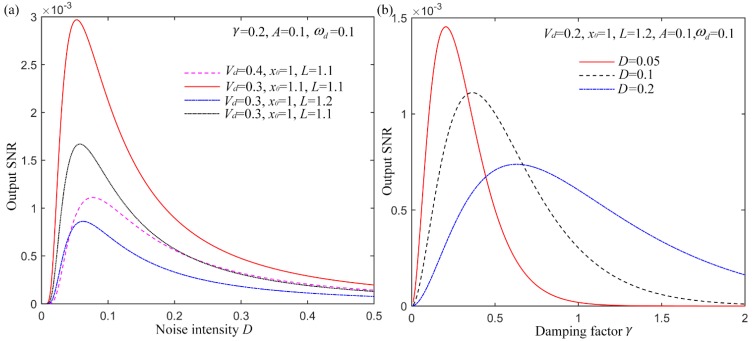
Theoretical output SNR of UPSR with different parameters, (**a**) output SNR *vs*. input noise intensity; (**b**) output SNR vs damping factor.

With the deduced analytical expression of the output SNR in the bi-stable stage of a UPSR system, the SNR *vs*. noise intensity *D* with different parameters are shown in Figure 3a. Set the damping factor γ=0.2, signal amplitude *A* = 0.1 and driving frequency $\omega_{d}=0.1$ here. Then we vary the noise intensity D from 0.001 to 0.5. Different system parameters of *V_d_*, *x_0_* and *L* are considered with output SNR displayed as Figure 3a. Apparently, the UPSR performs with the feature of the conventional SR system, which means that the SNR firstly increases, and then reaches a peak value, and finally decreases with the increasing of noise intensity. In addition, the system parameters also play crucial roles on the output SNR, which provides the possibility to tuning them for the optimal output. Subsequently, we fix the noise intensity at three different levels of 0.05, 0.1 and 0.2 but vary the damping factor from 0.01 to 2. The output SNR is then shown in Figure 3b with *V_d_* = 0.2, *x_0_* = 1, *L* = 1.2, *A* = 0.1 and ${\text{ω}}_{d}=0.1$. Results indicate that the SNR also shown a unimodal feature *vs.* the damping factor. And for different input noise intensity, the optimal γ stands at the different value, so with a certain input signal, the damping factor can be also optimized for the highest output SNR.

The above results provide the basic model and the SR phenomenon of an UPSR system. However, to apply the system to process engineering signals, some issues should be pre-considered: (1) Theoretical analysis is performed under the assumption of small parameter limitation. While in practical applications, the output cannot be obtained directly for the large parameter signal; (2) The analytic analysis is performed within a continuous time system. However, processing the digital signal in a discrete-time system is more convenient and efficient; (3) Equation (8) indicates the form of two first-order differential equations, which need to be solved numerically. Hence, to make the basic UPSR model able to detect weak digital signal, discrete fourth-rank Runge-Kutta method [26] is utilized to obtain the output discrete signal *x*[*i*] as Equation (21). In the formula, *h* is the calculation step interval. *t*[*n*] and *x*[*n*] represent the discrete forms of *t* and *x(t)*. s[i]+n[i] means the input driving signal mixed with noise. System parameters in Equation (7) and *h* act as the dominate roles that affect the system output. In fact, *h* often represents the reciprocal of sampling frequency *f_s_*. The majority of the theoretical studies in SR have been focused on low frequency and weak periodic signals interfered by noise. This can be explained by the fact that most of the studies were restricted by adiabatic approximation and linear response theory, where these parameters were assumed to be small [1]. To get rid of the restriction in numerical study of SR, a re-scaling frequency stochastic resonance is utilized here to shrink the parameters to a smaller scale by considering a rescaling ratio *R* proposed by Leng [27]. As a result, a new sampling frequency of *f_s_/R* with calculating step *h = R/f_s_* can be obtained. With the assistance of *R*, the characteristic frequency of input signal decreases from *f_0_* to *f_0_/R*, which contribute to get rid of the small parameter limitation. In the rest of this paper, the re-scaling ratio is fixed at a proper scale without tuning, as the research focuses on the influences of pinning potential parameters and damping factor in a UPSR system.

(21) $$\{\begin{array}{c} x\left[0\right]=x\left(t\left[0\right]\right);y\left[0\right]=y\left(t\left[0\right]\right)\;\\ k_{x1}=y\left[i\right]\\ k_{y1}=-V_{pin}^{'}\left(x\left[i\right]\right)-\gamma y\left[i\right]+\left(s\left[i\right]+n\left[i\right]\right)\\ k_{x2}=y\left[i\right]+\;\frac{h}{2}k_{y1}\;\\ k_{y2}=-V_{pin}^{'}\left(x\left[i\right]+\frac{h}{2}k_{x1}\right)-\gamma \left(y\left[i\right]+\;\frac{h}{2}k_{y1}\right)+\left(s\left[i\right]+n\left[i\right]\right)\;\\ k_{x3}=y\left[i\right]+\;\frac{h}{2}k_{y2}\;\\ k_{y3}=-V_{pin}^{'}\left(x\left[i\right]+\frac{h}{2}k_{x2}\right)-\gamma \left(y\left[i\right]+\;\frac{h}{2}k_{y2}\right)+\left(s\left[i+1\right]+n\left[i+1\right]\right)\\ k_{x4}=y\left[i\right]+hk_{y3}\;\\ k_{y4}=-V_{pin}^{'}\left(x\left[i\right]+hk_{x3}\right)-\gamma \left(y\left[i\right]+hk_{y3}\right)+\left(s\left[i+1\right]+n\left[i+1\right]\right)\\ x\left[i+1\right]=x\left[i\right]+\frac{h}{6}\left(k_{x1}+2k_{x2}+2k_{x3}+k_{x4}\right)\\ y\left[i+1\right]=y\left[i\right]+\frac{h}{6}\left(k_{y1}+2k_{y2}+2k_{y3}+k_{y4}\right) \end{array}$$

### 2.3. Weak Signal Detection Scheme

Before the novel weak signal detection strategy is proposed, a criterion to evaluate the output signal and system performance is essential, because all the parameters need to be optimized for the best matching system and optimal output. SNR is considered here which is defined as the power spectral density of the driving signal divided by the sum of all power spectral density of background noise or interference. With the calculated output of *x*[*n*], the output SNR can be obtained by calculating the power spectrum via fast Fourier transform (FFT) numerically with result of:

(22) $$\text{SNR}=10{\text{log}}_{10}\frac{{\text{A}}_{\text{s}}}{\sum_{\text{i}=1,\text{i}\neq \text{s}}^{\text{N}/2}{\text{A}}_{\text{i}}}$$

In the formula, N is the data length for a discrete signal and *A_s_* means the power of driving frequency in the spectrum. $\sum_{\text{i}=1,\text{i}\neq \text{s}}^{\text{N}/2}{\text{A}}_{\text{i}}$ indicates the total power of noise. In this case, a higher SNR shows that the mix signal contains less interference and the driving frequency is easier to be distinguished.

**Figure 4:**
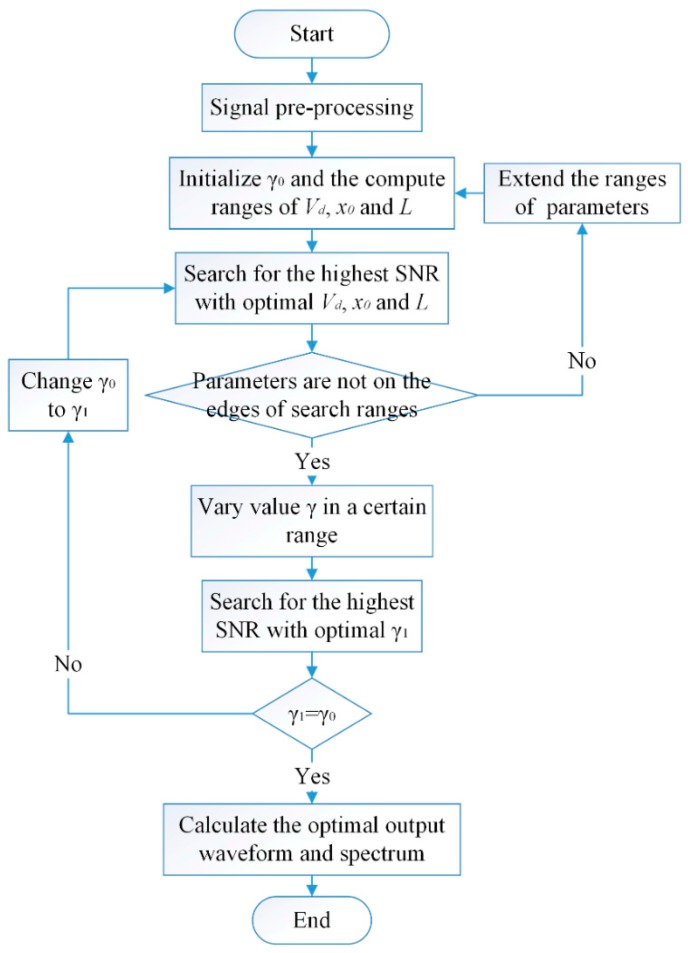
Weak periodic signal detection scheme based on UPSR.

In fact, for the SR system, the output SNR comes to the peak value with a certain input noise intensity. So the optimal noise intensity can result in the optimal output. However, tuning the noise intensity seems impossible for an acquired mixed signal whose noise and driving signal are all fixed. As a result, from another point of view, the system parameters can be optimized to be adapted to the input noise level. Then the optimal output SNR can be also obtained. In order to gain the highest output SNR by adjusting system parameters jointly, a strategy to detect the weak periodic signal with background noise based on UPSR is synthetically presented as shown in Figure 4. As the global search among 4-dimension ($V_{d}$, $x_{0}$, *L* and γ) is not quite efficient, the damping factor is isolated for optimization and the strategy can be separated into five steps. The detailed procedure is described as follows:

(1) *Signal pre-processing*. The signal is pre-processed with techniques of filtering part of the noise that locates in the uncorrelated bandwidth, and extracting the driving frequency by calculating its envelope signal where the driving frequency is modulated to a high level.
(2) *Parameter initialization*. Fix the damping factor of ${\text{γ}}_{0}$ and initialize the calculating ranges of $V_{d}$, $x_{0}$ and *L*.
(3) *Optimization of* $V_{d}$, $x_{0}$ and *L*. Firstly, calculate the power spectrum of the output waveform and obtain its SNR according to Equations (21) and (22). Then search for the maximal SNR over varying variables $V_{d}$, $x_{0}$ and *L*, and obtain the optimal parameters combination corresponding to the peak value. Finally, check that whether the values have reached the edges of searching ranges. If so, go back to step 2 and extend the searching ranges. Otherwise, go to next step.
(4) *Optimization of* γ. With the temporarily optimized parameters of $V_{d}$, $x_{0}$ and *L*, change the value of γ in a reasonable range and calculate the output SNR with different γ. Search for the highest SNR and the corresponding ${\text{γ}}_{1}$. If ${\text{γ}}_{0}={\text{γ}}_{1}$, go on with the next step. If not, go back to step 2 by replacing ${\text{γ}}_{0}$ with the new optimal ${\text{γ}}_{1}$.
(5) *Acquisition of optimal output*. Obtain the optimal output waveform as the detected weak signal with the optimized parameters. The periodic signal is then detected successfully and noise is weakened apparently. Calculate its power spectrum for the identification of the driving frequency. By these operations, the feature in weak periodic signal can be recognized clearly, which is also beneficial to the rotating machine fault diagnosis.

## 3. System Performance of UPSR

### 3.1. Output of UPSR

Firstly, with the proposed UPSR model and numerical solution method, a sinusoidal signal with Gauss white noise of −5 dB (added by AWGN in MATLAB R2014b) is treated as the input signal of Equation (7):

(23) $$s\left(t\right)=Acos\left(2\text{π}f_{d}t+{\text{φ}}_{0}\right)$$

**Figure 5:**
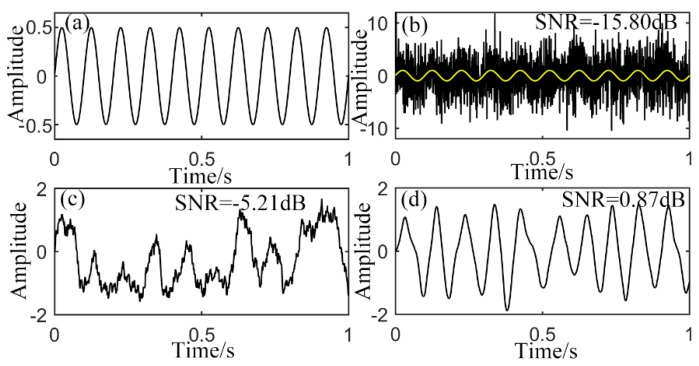
Output of sinusoidal input, (**a**) pure signal; (**b**) input with noise; (**c**) output of CSR with presetting parameters; (**d**) output of UPSR with presetting parameters.

Here the amplitude *A* is set at 0.5 with driving frequency $f_{d}=10$ Hz. Initial phase is ignored ($\varphi_{0}=0$). The pure signal is shown in Figure 5a and the yellow curve in Figure 5b with sampling frequency of 2000 Hz. Figure 5b represents the mixed signal, where the input SNR is calculated to be −15.80 dB according to Equation (22). For comparison, the over-damped CSR system with polynomial potential model denoted by the equation as below is taken into consideration:

(24) $$\frac{dx}{dt}=-V^{\prime }\left(x\right)+s\left(t\right)+n\left(t\right)=ax-bx^{3}+s\left(t\right)+n\left(t\right)$$

Where *a* and *b* are the parameters of bi-stable potential corresponding to Equation (2). s(t)+n(t) still means the input signal as Equation (1). Analyze the signal in Figure 5b with this system and the output is shown in Figure 5c. System parameters are preset at *a* = 0.8 and *b* = 1. The re-scaling ratio used here is 50 (it maintains the same value in the rest of this paper if not otherwise mentioned). According to the output signal, ten periods during 1 seconds is extracted to some extent, but result is not quite satisfying and the noise of high frequency is still frustrating. Then the simulated signal is processed by the proposed UPSR with presetting parameters of $V_{d}=8$, $x_{0}=1$, *L* = 1.3 and γ=0.4. The output is displayed in Figure 5d where the waveform is quite neat without much noise and it is also closer to the pure input signal in Figure 5a.

**Figure 6:**
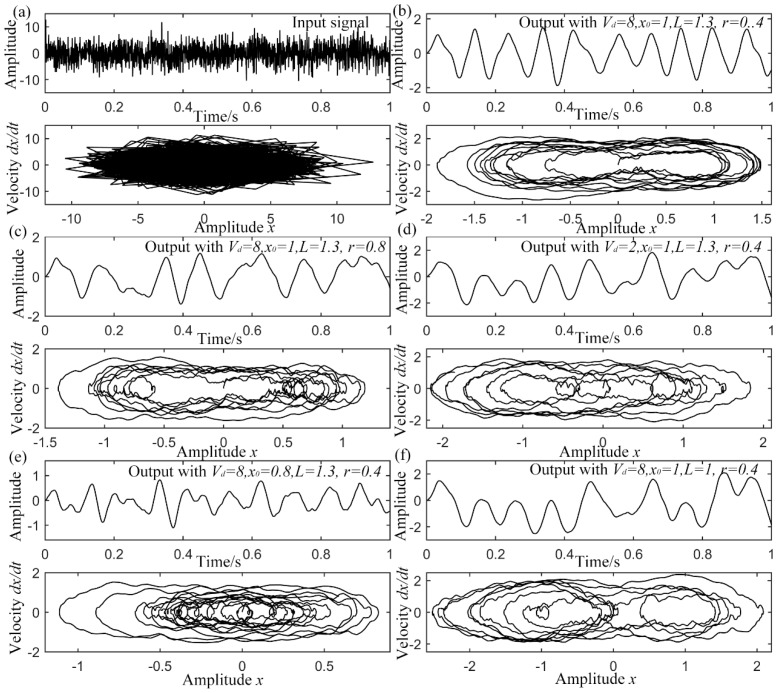
Output of UPSR with sinusoidal input and different system parameters, (**a**) original input signal waveform and phase diagram; (**b**) output with of $V_{d}=8$, $x_{0}=1$, *L* = 1.3 and γ=0.4; (**c**) output with of $V_{d}=8$, $x_{0}=1$, *L* = 1.3 and γ=0.8; (**d**) output with of $V_{d}=2$, $x_{0}=1$, *L* = 1.3 and γ=0.4; (**e**) output with of $V_{d}=8$, $x_{0}=0.8$, *L* = 1.3 and γ=0.4; (**f**) output with of $V_{d}=8$, $x_{0}=1$, *L* = 1 and γ=0.4.

To evaluate the performance of UPSR, some simulations have been carried out by employing the same input in Figure 5b but system parameters are different. The outputs and their corresponding phase diagram (PD) are exhibited in Figure 6. In Figure 6a, original trajectory is totally destroyed by the disordered noise. Figure 6b shows the same output as Figure 5d and its PD. Well-arranged trajectory is found and the periodicity is apparent. When the damping factor is added to 0.8 in Figure 6c, the system performance becomes bad as a too large γ makes it difficult for the particle to follow pace of the periodic input and more low-frequency components occur. On the other hand, if the damping factor is too small, it is hard for the system to be stable with outside excitation and more severe oscillation comes up. So more high-frequency components occur. The same phenomenon appears for the other parameters. The $V_{d}$ decreases to 2 in Figure 6d, $x_{0}$ decreases to 0.8 in Figure 6e and *L* decreases to 1 in Figure 6f. Worse performances of system all come up. This can be explained qualitatively. In fact, a smaller $V_{d}$ leads to a lower and milder potential wall. It is easier for the particle to jump out of it and less probable be rebounded back. So low-frequency interference plays the main role. On the other hand, larger $V_{d}$ leads to a higher and steeper potential wall. The oscillation between the two wells becomes fiercer and high-frequency components occur. The role of $x_{0}$ and *L* affect the equilibrium positions and potential barrier that the particle needs to overcome. A small $x_{0}$ with a large *L* generates a mono-stable potential model as described in Section 2.1 or the barrier is quite low in bi-stable one. The equilibrium positions get closer with each other at small $x_{0}$. In this case, it is quite easy for the particle to transmit between the two wells and fiercer transmission leads to output with components of higher frequencies. Conversely, a large $x_{0}$ with a small *L* results in high barrier and long distance between the equilibrium positions. This makes it tough for the particle to surmount the barrier and reach the other well. The particle needs more time to collect sufficient energy, so the period becomes long. On the circumstance, low-frequency interference makes the output unsatisfying as shown in Figure 6f. The particle even can’t overcome the barrier, which is indicated by the position of 0. In its PD, trajectory moves round one of the wells some times. According to the above description, when regarded as a selected passing filter, the parameters of UPSR system play the dominant roles, where larger or smaller ones can result in deflected output with whether high- or low- interference frequencies. In other words, all the parameters can be optimized for the most precious output signal and meanwhile, the weak periodic signal can be successfully enhanced.

**Figure 7:**
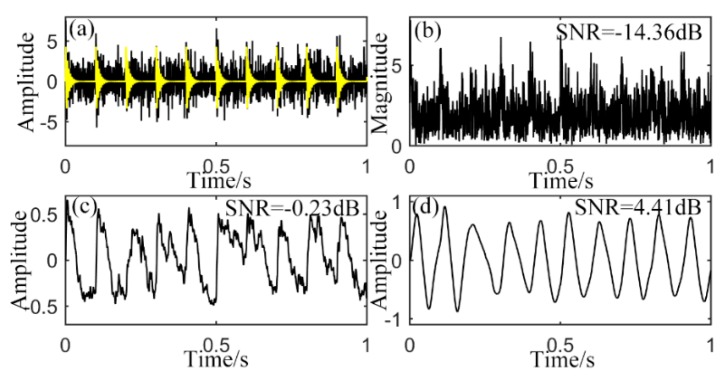
Output of unilateral attenuation impulse input, (**a**) original signal with noise; (**b**) envelope; (**c**) output of CSR with presetting parameters; (**d**) output of UPSR with presetting parameters.

In practical application like rotating machine fault diagnosis, there is another widely existing signals called unilateral attenuation impulse [28,29,30,31,32,33]. Rotating machinery response is often characterized by the presence of periodic impulses modulated by high-frequency harmonic components. Its waveform is shown as the yellow curve in Figure 7a with *A* = 4, attenuation rate *d* = 100, characteristic frequency *f_0_* = 10 Hz and intrinsic frequency *f_m_* = 200 Hz. Sampling frequency is still 2000 Hz. Intrinsic frequency is the basis of demodulated resonance, which is determined by a mass of factors, such as vibration of inner race, outer race, balls and sensors in the situation of bearing vibration signal. But what we really concern is the characteristic frequency modulated by the intrinsic frequency to a higher level. Frustratingly, the frequency is often overwhelmed by background noise, which makes it difficult to be recognized. Many researchers make their efforts to the de-noising work of such signal. The black curve in Figure 7a means the impulsive signal with noise of −3 dB added. Figure 7b shows its envelope signal calculated by Hilbert transform (HT) which is unilateral with SNR of −14.36 dB according to Equation (22). In order to have the simulated impulsive signal, it is generated according to the equation [32,33] where the initial phase is ignored:

(25) $$s_{impulse}\left(t\right)=Asin\left(2\text{π}f_{m}t\right)\cdot exp\left(-d\cdot \text{mod}\left(t,T_{0}\right)\right)$$

In the formula, *A* means the signal amplitude, *f_m_* is the intrinsic frequency, *d* indicates the attenuation rate and $f_{0}=1/T_{0}$ is the characteristic frequency. mod(a, b) is the remainder of *a* divided by *b* which controls the impulses appear periodically. The envelope signal in Figure 7b is then analyzed with both CSR and UPSR systems. Figure 7c shows the output signal of CSR with parameters of *a* = 0.8 and *b* = 10. The output SNR is calculated to be −0.23 dB. Although the periodicity is revealed, the output still suffers from noise, which makes the result defective. For the output of UPSR with system parameters of $V_{d}=8$, $x_{0}=0.8$, *L* = 1.1 and γ=0.4, it performs almost perfectly with the periodic signal obviously extracted and little noise remains. Ten cycles during a second are apparently indicated, which means frequency of 10 Hz. Output SNR is also enhanced to 4.41 dB. This comparison shows that the proposed method is also applicable to unilateral attenuation impulses detection, which can be applied in rotating machine defect detection.

### 3.2. Influence of System Parameters

**Figure 8:**
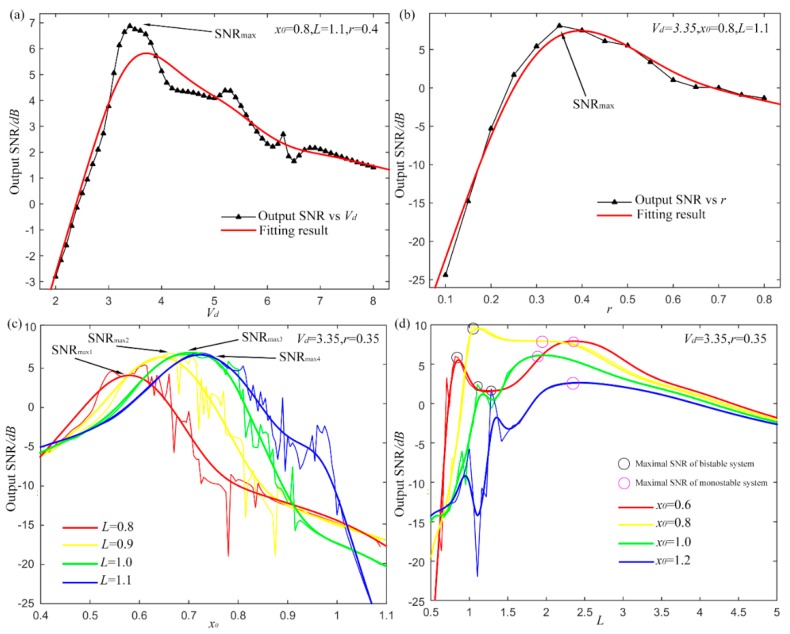
Influences of system parameters, (**a**) output SNR with different $V_{d}$; (**b**) output SNR with different damping factor γ; (**c**) output SNR *vs*. $x_{0}$ with different *L*; (**d**) output SNR *vs*. *L* with different $x_{0}$.

Subsequently, it is imperative to evaluate the system performance with different system parameters to certify the feasibility and effectiveness of parameters optimization. A sinusoidal signal as in Equation (23) with amplitude *A* of 0.5 and driving frequency $f_{d}=10\;$Hz is utilized here. The initial phase is still ignored (${\text{φ}}_{0}=0$). Gauss white noise of −8 dB is added by AWGN. Sampling frequency $f_{s}=2000\text{ Hz}$ and rescaling ratio is set at 50 as last subsection. Figure 8 shows the impact of system parameters.

The parameter $V_{d}$ firstly varies from 2 to 8 with the others fixed: $x_{0}=0.8$, *L* = 1.1 and γ=0.4. Then the output SNRs with different value of $V_{d}$ are calculated out and Figure 8a shows the results. Not surprisingly, SNR increases firstly and then decrease with the increasing of $V_{d}$. A distinct peak value occurs at a certain value of $V_{d}$. The red curve in the figure shows the fitting result. Similarly, the output SNRs with different damping factor γ also perform single peaked as Figure 8b with fixed parameters of $x_{0}=0.8$, *L* = 1.1 and $V_{d}=3.35$. As a result, the optimal value of γ for the highest output SNR is also found. The results in these two figures indicate the probability to optimize the parameters $V_{d}$ and γ for UPSR system.

Then $x_{0}$ and *L* are analyzed together as their value relation determines the equilibrium and barrier height of pinning potential model. In Figure 8c, $x_{0}$ changes from 0.4 to 1.1 with different *L*. Optimized $V_{d}$ and γ are adopted with value of 3.35 and 0.35. Four curves of different colors show the output SNRS and the fitting results under different *L*. For each *L*, the optimal *x_0_* has the different value. This shows the coupling of the two parameters to some extent. Besides, when *L* gets larger, the optimal value of $x_{0}$ also increases. According to the result in Equation (6), a larger *L* leads to a larger $x_{0}$, which can make the system perform bi-stable. But the larger *L* is not necessarily the better. In Figure 8d, *L* varies from 0.5 to 5 with four different $x_{0}$. Different from the one peak curve in Figure 8c, the results here display a double-peak feature. The two peaks occur at a small *L* and a large *L* separately as rounded by the black and pink circles in Figure 8d. In fact, the two peaks come up at the different stage with *L* increasing. The black ones mean the maximal SNR of bi-stable stage and pink ones for the mono-stable stage. While it is indeterminate that whether the optimal output stands in the bi-stable or mono-stable stage. In this case, the bi-stable stage of yellow curve provides the optimal output with $x_{0}=0.8$ and *L* = 1.05. The results show that the proposed UPSR can realize both the bi-stable and mono-stable systems by tuning $x_{0}$ and *L*, which cannot be achieved by the CSR system. Sometimes, the optimal result may come up with a mono-stable system, which is influenced by the calculating step *h* in Equation (21). It is really affected by the re-scaling ratio. A small re-scaling ratio leads to a small *h*. In this circumstance, the particle cannot restore enough energy and jumping over the barrier seems impossible. This is also an intuitive explanation that why the bi-stable system suffers from the small parameter restriction that only signals with small driving frequency work well. On the contrary, a large re-scaling ratio leads to a large *h* and enough energy is provided. For UPSR, however, even if the re-scaling ratio is fixed at a certain level, for driving signal with large frequency, the system can be adjusted to mono-stable to adapt the input signal and provide satisfying output, which is further demonstrated in next section. The feature makes the proposed method possess stronger adaptability and robustness for different kinds of signals.

### 3.3. Performance with Different Input Signals

In last subsection, the impacts of system parameters on output have been analyzed. More meaningfully, the frequency response and output performance *vs*. input noise are revealed, as such metrics are rather critical in practical signal processing application.

#### 3.3.1. Performance *vs*. Driving Frequency

**Figure 9:**
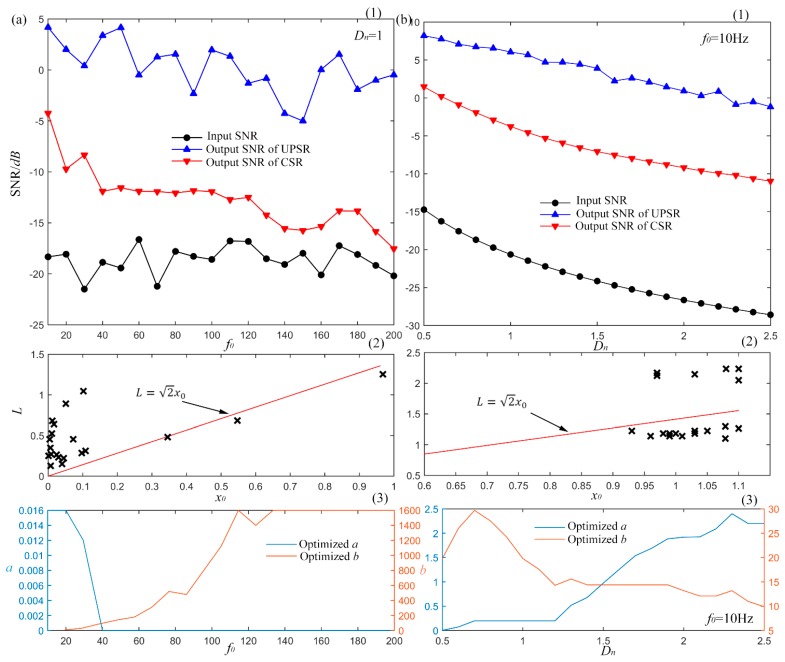
System performance with different input signals, (**a**) system performance with different driving frequency and the optimized parameters; (**b**) system performance with different input noise and the optimized parameters.

Firstly, the simulated signal is still sinusoidal as Equation (23). Amplitude *A* is fixed at 0.5 with noise of −8 dB added, sampling frequency $f_{s}=2000\text{ Hz}$ and rescaling ratio equals to 50. Different from the signal in Section 3.2, the driving frequency changes from 10 Hz to 200 Hz with interval of 10 Hz. Each frequency *f_0_* generates a certain signal. When regarded as the input of SR system, they provide different output signals and SNRs as shown in Figure 9a. For each set of signal with different driving frequencies, the optimal output of UPSR is searched as shown by the blue curve in Figure 9a(1) according to the scheme in Figure 4. For comparison, the peak output SNR of a CSR system is also achieved as the red curve in Figure 9a(1). The black line denotes the original input SNRs. Because of the randomness of the Gauss white noise and the numerical calculating error, it fluctuates around a certain value, which may be uninfluential for the results. The figure indicates that the UPSR performs much better than the CSR system, which consistently provides higher output SNR in all frequency band. What’s more valuable is that the UPSR can still generate effective output with high driving frequency. This indicates that the UPSR possesses better frequency response than the CSR.

Figure 9a(2) and Figure 9a(3) show the parameters optimization results for UPSR and CSR respectively. In Figure 9a(2), the coordinate values of each cross point stand for the values of optimized ($x_{0}$, *L*) with different driving frequencies. The red line shows the critical condition of bi-stable and mono-stable states. Area over the line is the mono-stable potential and area under the line stands for the bi-stable state. The three points under the line show the first three results of frequency 10 Hz, 20 Hz and 30 Hz frequency. The optimal $x_{0}$ are large relatively. This can be explained by the fixed re-scaling ratio of 50. It makes the equivalent driving frequency of 0.2 Hz, 0.4 Hz and 0.6 Hz. They can be regarded as the small parameters, which make the particle accumulate enough energy to jump over the barrier in one period and the SR effect comes up with the bi-stable potential model. But with the increasing of *f_0_*, the equivalent driving frequency also increases and energy that the particle gains in one period in not enough to overcome the potential barrier. So the optimal state comes to the mono-stable area with small $x_{0}$ relatively and the SR effect occurs with a mono-stable potential model. This shows the adaptability and robustness of UPSR which can be converted between bi- and mono- stable states conveniently for different input signals. However for the CSR system as Equation (24), the mono-stable state cannot be reached. The optimization results in Figure 9a(3) indicate that with the increasing of driving frequency, parameter *a* approaches to an extreme small value while *b* approaches to a large one until they reach the boundary, which produce a bi-stable potential model with smaller and smaller barrier in fact. Because the energy of the particle in one period becomes lower and lower, which can only conquer lower and lower potential barrier. In terms of input signal with high driving frequency, the SR effect fails to appear in CSR system as a matter of course. The results verify the superiority of UPSR system, which performs better in high frequency band owing to its potential diversity.

#### 3.3.2. Performance *vs*. Input Noise Intensity

Subsequently, sinusoidal signal is simulated with driving frequency of 10 Hz and different noise amplitudes from 0.5 to 2.5 with steps of 0.1. All the other parameters are same as the previous subsection. We calculate the output SNR of different sets of signals. Then the results are displayed in Figure 9b with the blue line standing for the UPSR output and red line representing CSR output in Figure 9b(1). The black one still shows the input SNR, which falls with the noise increasing noise. Output SNRs of UPSR are found to be higher than the ones of CSR at whole levels of input noise. Besides, with the increasing of input SNR, the declining trend of UPSR is slower than the CSR, which indicates that the proposed system performs better than the CSR system confronting with heavier background noise. Therefore, UPSR possesses better anti-noise capacity and provides more satisfying output signal with higher input SNR.

The optimization results of the system parameters are shown in Figure 9b(2,3). For UPSR, $x_{0}$ and *L* are still shown. By analyzing the optimal combination, mono-stable model is more suitable for the input with lower noise, while bi-stable performs better with higher input noise. In Figure 9b(2), cross points locating in the mono-stable area are the parameters for input with lower noise, but points in the bi-stable area are just the opposite. These are also noticeable as too little noise makes it tough for the particle to traverse the barrier. So the barrier disappears during the optimization procedure of UPSR in the low-noise stage. While for the CSR system, only the height of barrier can be adjusted with impossible achievement of the potential model transformation. To acquire optimal performance with the increasing of noise, the barrier of CSR system should be increasingly higher, which is realized by the falling of *a* and rising of *b*. Thus the results in Figure 9b(3) present such a variation pattern. Consequently, the potential diversity and parameter sensibility endow the superiority of the proposed strategy in signal detection with different input noise.

## 4. Engineering Application

**Figure 10:**
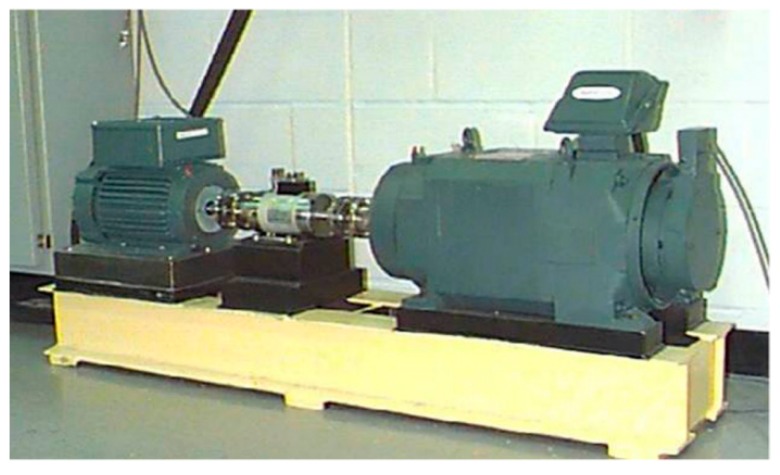
Bearing test rig of CWRU.

To verify the availability and effectiveness of the proposed UPSR method, different sets of bearing data with kinds of defects are analyzed for fault diagnosis of rotating machine according to the scheme as shown in Figure 4 in this section. As a comparison, these data are also addressed by the CSR method. The signals utilized here are taken from the Bearing Data Center in Case Western Reserve University (CWRU) acquired by using an experimental setup as shown in Figure 10. The test rig consists of a 2 hp motor (left), a torque transducer/encoder (center), a dynamometer (right), and control electronics (not shown). Test bearings employed here are the deep groove ball bearings with 6205-2RS JEM SKF type and the geometry parameters are provided in Table 1. Vibration data were collected using accelerometers, which were attached to the housing with magnetic bases, under sampling frequency of 12 kHz for driving end bearing experiments. Single point faults were set on the test bearings separately at the outer-, inner- raceway and rolling element using electro-discharge machining with different sizes. In fact, the periodical impulses emerge in the acquired vibration signal when a defect occurs in a bearing. Thus, the fault types can be estimated by analyzing the impulse period [34,35]. The characteristic frequencies (impulses frequency) for the fault information are calculated based on the fault type and rotating speed of motor accordingly [36]:

(26) $$\{\begin{array}{c} f_{BPFO}=\frac{Zf_{r}}{2}\left(1-\frac{D_{1}}{D_{2}}cos\alpha \right)\\ f_{BPFI}=\frac{Zf_{r}}{2}\left(1+\frac{D_{1}}{D_{2}}cos\alpha \right)\\ f_{BSF}=\frac{f_{r}D_{2}}{D_{1}}\left(1-{\left(\frac{D_{1}}{D_{2}}cos\alpha \right)}^{2}\right) \end{array}$$

**Table 1:** Specification of the bearing from CWRU.

Inside Diameter (Inch)	Outside Diameter (Inch)	Pitch Diameter (Inch)	Ball Diameter (Inch)	Number of the Rollers
0.9843	2.0472	1.537	0.3126	9

In the equation, *Z* is the number of rolling elements and *f_r_* stands for the rotating frequency. *D_1_* and *D_2_* are the diameters of one rolling element and the pitch diameter of the bearing. α is the contact angle. Characteristic frequencies of outer-race, inner-race and rolling element defect are denoted by $f_{BPFO}$, $f_{BPFI}$ and $f_{BSF}$ respectively. Three sets of signals with different fault conditions are selected for the following analysis. They are collected from bearings with different fault sizes and rotating speeds. The fault related parameters of fault sizes (depth and width) and rotating speeds as well as calculated fault frequencies according to Equation (26) are shown in Table 2.

**Table 2:** Parameters of bearings with different defect types.

Defect Type	Fault Size (D×W, Inch×Inch)	Rotating Speed (RPM)	Fault Frequencies (Hz)
Outer-race defect	0.011×0.007	1772	105.9
Inner-race defect	0.011×0.014	1750	157.9
Rolling element defect	0.011×0.021	1730	135.9

**Figure 11:**
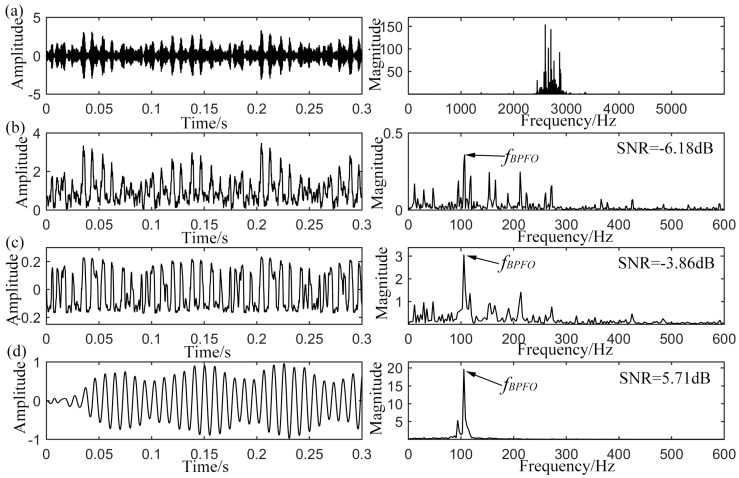
Analyzed results of the outer-race defective signal from CWRU, (**a**) original input signal with its spectrum; (**b**) envelope signal with its spectrum; (**c**) output of CSR with its spectrum; (**d**) output of UPSR with its spectrum.

### 4.1. Outer-Race Defect Detection

Firstly, Figure 11a shows the waveform and power spectrum of the bearing vibration signal with outer-race defect. According to Table 2, the ball passing frequency over the defect position, *f_BPFO_*, is calculated to be 105.9 Hz with approximate rotating speed of 1772 rpm. In the time domain of Figure 11a, the defect information cannot be clearly identified. The frequency domain indicates obvious elevated amplitude on the structure resonance band making the fault frequency modulated to high-frequency band among 2500 Hz to 3500 Hz. However, this is not indicative of structural defect. Get its envelope signal by demodulating it via Hilbert transform (HT). The time and frequency domain results are displayed in Figure 11b with input SNR of −6.18 dB. It can be seen from the power spectrum that the defect-induced frequency component *f_BPFO_* can be pointed out, while the noise components around *f_BPFO_* make the diagnosis result be hardly seen. Following on, the output results of the CSR system are exhibited in Figure 11c with optimized parameters of a=0.005 and b=160, where the rescaling ratio is fixed to be 200 and the same value is adopted for the later analysis of inner-race and rolling element defects. It is found that the *f_BPFO_* can be pointed out in the power spectrum and the SNR is improved to −3.86 dB. But some noise components around the *f_BPFO_* still interfere the judgment. Finally, the proposed UPSR strategy is utilized to detect the weak defect-induced impulses. The system parameters are optimized to $V_{d}=11.6$, $x_{0}=0.48$, L=1.73 and γ=0.18. The results illustrated in Figure 11d indicate that the component *f_BPFO_* = 105.5 Hz (within acceptable error range) has been further enhanced and stands out when compared to the former results. The output SNR is improved to 5.71 dB which provides a quite satisfying output and reliable diagnosis basis. As a result, the proposed strategy shows an enhanced capacity in detecting the weak periodic signal, when compared to the CSR method.

### 4.2. Inner-Race Defect Detection

**Figure 12:**
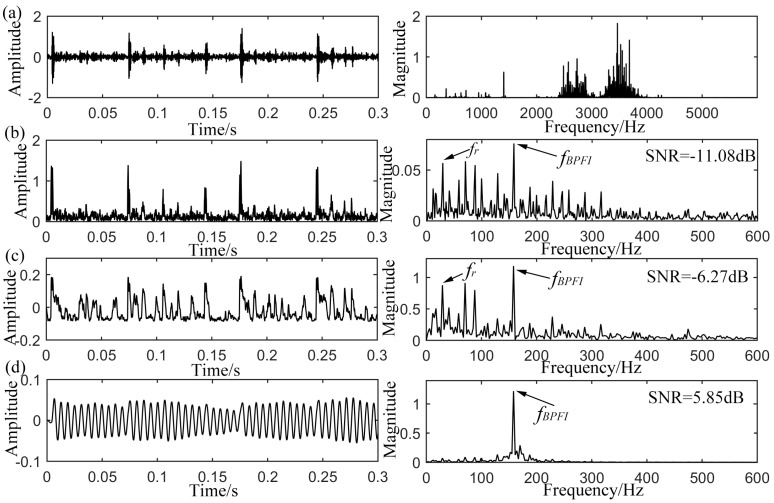
Analyzed results of the inner-race defective signal from CWRU, (**a**) original input signal with its spectrum; (**b**) envelope signal with its spectrum; (**c**) output of CSR with its spectrum; (**d**) output of UPSR with its spectrum.

Furthermore, the inner-race defective signal with approximate rotating speed of 1750 rpm is analyzed again. The fault frequency is calculated to be 157.9 Hz according to the geometry in Table 1. The raw and envelope signals with their corresponding power spectrums are shown in Figure 12a,b respectively. When demodulated, the *f_BPFI_* can be pointed out in the envelope spectrum in Figure 12b. However, the noise interferences as well as the component of rotating frequency *f_r_* are still obvious in both time and frequency domains. The input comes up with SNR of −11.08 dB. For the optimal output of CSR method in Figure 12c with a=0.075 and b=186, the majority of high-frequency noise is found to be suppressed. However, some low-frequency noise components (e.g., *f_r_*) are also amplified unexpectedly and stand frustratingly, although with an improved SNR of −6.27 dB. In relative terms, the result of the proposed method is more serviceable as shown in Figure 12d. Compared to the original envelope signal, clearer period can be recognized from the output time domain waveform. The power spectrum indicates that the noise components have been suppressed while the fault characteristic frequency *f_BPFI_* = 158.2 Hz is distinctly highlighted. With the optimal combination of system parameters $V_{d}=15.8$, $x_{0}=0.35$, *L* = 1.40 and γ=0.18, the output SNR gets to 5.85 dB. This confirms the effectiveness and superiority of the proposed method again in extracting the periodic signal for mechanical fault information.

### 4.3. Rolling Element Defect Detection

**Figure 13:**
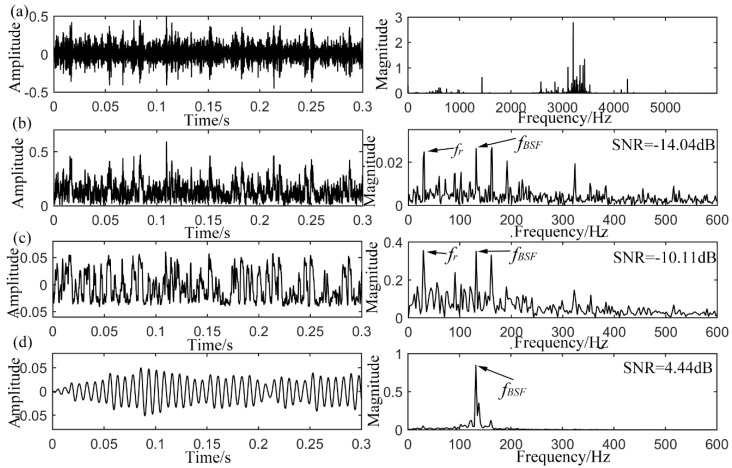
Analyzed results of the rolling element defective signal from CWRU, (**a**) original input signal with its spectrum; (**b**) envelope signal with its spectrum; (**c**) output of CSR with its spectrum; (**d**) output of UPSR with its spectrum.

Finally, the rolling element defective signal is analyzed by different methods, and the results are shown in Figure 13 synthetically. With a rotation speed of 1730 rpm, the fault frequency locates at 135.9 Hz. The original signal and its spectrum in Figure 13a offer no available information for the confirmation of defect type, except for a resonance band according to the frequency domain. In its envelope signal in Figure 13b, impulses emerge in the waveform, but the noise interference is so strong that the impulse intervals of impulse occurring cannot be confirmed. In the envelope spectrum, the defect-induced frequency *f_BSF_* is extracted with SNR of −14.04 dB, while it is relatively weak when compared to the low frequency noises especially the rotating frequency of *f_r_* and some other nearby interferences. The output of CSR system is shown in Figure 13c with its waveform and spectrum. The optimal parameters are a=0.001 and b=360. It is found that the defect-induced frequency *f_BSF_* is enhanced in the power spectrum, but some unexpected lower frequency noises are also synchronously amplified together. The output SNR is calculated to be −10.11 dB, which is already amplified with 3.93 dB. Then the proposed UPSR is employed to process the same signal with rolling element defect and the output is displayed in Figure 13d. Almost a single frequency is exhibited in the spectrum and the signal shows well-arranged waveform and distinct intervals. The highlighted component with SNR of 4.44 dB in the spectrum just indicates the fault characteristic frequency *f_BSF_* of 135.2 Hz. The final optimal parameters combination is $V_{d}=15.4$, $x_{0}=0.42$, *L* = 1.72 and γ=0.13. Consequently, the proposed UPSR shows an improved effect in detecting the weak signals in comparison with the CSR method.

### 4.4. Discussions

The above sections present the system performance with both simulated and experimental signals. Output SNRs of different kinds of defect bearing signals are listed in Table 3 for comparison as well as the raw envelope signal and outputs of CSR system. The results and distinctions indicate that the proposed UPSR method possesses superiority in extraction of the weak defect-induced impulses under different working conditions for rotating machine fault diagnosis when compared to the CSR system. Some discussions on the merits and prospects of UPSR system are presented as follows.

(a) The damping factor involvement provides a secondary filtering effect for the SR system. The UPSR can perform as an underdamped or approximate over-damped system by tuning the damping factor value. It ensures the system to provide higher output SNR with less interference components and better universality to different kinds of input signals with different noise levels. Choosing a proper damping factor makes the underdamped SR perform better in enhancing weak signal due to the secondary band-pass filtering effect, which acts as a specific selective non-linear filter.
(b) The simulation results of the system performance with different input signals indicate the better anti-noise ability and frequency response in detection of signals with strong background noise. Transferring the noise energy to the target frequency makes it beneficial to detect the weak signals that are involved in the noise bandwidth. All the system parameters can be optimized more accurately and easily for the best combination and the highest output SNR due to its parameters sensibility. The three features (barrier height, equilibrium position and wall steepness) of the potential well play dominant roles on the performance of SR. For the CSR model, they are determined by only two parameters, *a* and *b*, which indicates that the parameters are not independent or one feature sensitive, but for the UPSR system, the different parameters possess definite duties. Thus, the optimal potential model to enhance the periodic weak signal can be accurately designed. When searching all the parameters referring to the global search method, the efficiency of the UPSR system with three parameters is obvious lower than that of ordinary cubic potential model, which has only two dimensions, so the efficiency of the optimization procedure for system parameters may be further improved by referring to the progressive searching algorithm (like genetic algorithm) in the future to bring down the consumption of the exhaustive search.
(c) Traditional bi-stable SR theory is under the assumption that the frequency of the periodic signal is smaller than one, so the rescaling ratio is utilized here to lower the equivalent driving frequency and enlarge the calculating step h in Equation (21). For the CSR system, the calculating step is strictly confined, which affects the output severely, because the CSR system always acts as a bi-stable model and jumping over the barrier for the particle is essential, but for systems with relatively small h, the energy in one period is not enough to overcome the barrier. On the other hand, too large an h value has the consequence of too much high frequency noise. However the proposed UPSR system weakens the restriction of the calculation step. Whenever the signal contains high or low driving frequency or background noise, the system can optionally transform between two states. A mono-stable one comes up for the signals with relatively large driving frequency just as the three cases in engineering application. In other words, the model can be adjusted to adapt to different scales of calculating step. This feature makes the system more adaptive and robust to various kinds of input signals and less dependent on the small size of signals.
(d) The output SNR in Equation (22) is employed here as the detection and optimization criterion. However, it can be improved ultimately by optimizing the decision like replacing the SNR by mutual information, Fisher information, correlation or other discrimination indexes [37]. Much attention is also paid to the different decisions. Sequential detection performance via SR was evaluated in terms of the Neyman-Pearson and Bayesian criteria [38]. The pertinent receiver operating characteristics were compared with those of the known statistically optimum detector using extensive Monte Carlo simulations [39]. In [40], it was mentioned that the naive sample mean detector is outperformed, in terms of error probability, by the optimal likelihood ratio test. Besides, other detection strategies like Bayesian, minimum error-probability, Neyman-Pearson, and minimax detectors were all investigated in [41]. Appreciably, most of the cases refer to the framework of statistical detection theory. In our study at the current stage, independent detection and diagnosis is considered, which means the diagnosis work at the current stage of our work is made independently, but not statistically. While the statistical analysis and diagnosis for rotating machine is preferred for more accurate results, averting the limit of optimal Neyman-Pearson testing technique. We also would like to avoid the problem of counterintuitive “improvements by noise” by improving the detection strategy employing the novel and effective criteria in the future.
(e) Although it displays the enumerated merits, the system still needs to be further studied to develop its physical theory. In spite of the weakened impact of the calculation step, its role to the final optimization results still needs to be explored. Besides, inconformity of output amplitude is found in our study, especially when the system is mono-stable. Investigations on the formation mechanism and strategy to conquer this matter are also needed. Besides, the application of UPSR system differs from the theoretical SR whose optimal condition is achieved by tuning the input noise intensity. The existence of a better detection when noise increases should not be underestimated, so we would like to investigate novel noise tuning methods to apply the new SR system in signal de-noising and weak signal detection in the future.

**Table 3:** The performance of different methods in processing defective bearing signals.

Defect Type	Envelope Signal SNR (dB)	Output SNR of CSR (dB)	Output SNR of UPSR (dB)
Outer-race defect	−6.18	−3.86	5.71
Inner-race defect	−11.08	−6.27	5.85
Rolling element defect	−14.04	−10.11	4.44

## 5. Conclusions

This paper investigates a new potential model to realize underdamped SR system. A weak periodic signal detection strategy based on UPSR is further proposed and explained in detail by exploring the influences of system parameters and performances with different input signals. Engineering applications to rolling element bearing fault diagnosis are analyzed. Results indicate that the proposed method can be implemented by tuning the system parameters ($V_{d}$, $x_{0}$, *L* and γ), ignoring the calculating step. Then the optimal system can be designed accurately for the highest output SNR and a satisfying output signal. The superiority of the proposed method in comparison with a CSR method is illustrated by numerical simulation analysis and engineering applications. The results show that the damping factor provides a higher output SNR and ensures the system perform as a more powerful filter. A unique potential model makes it more efficient and effective to adjust the system parameters. Better frequency response and anti-noise performance than the CSR system are investigated. The potential diversity makes the SR effect come up more easily and weakens the limitation of small parameters on SR system. Considering all its merits, the method can be expected to be widely applied in weak periodic signal detection, especially in the area of rotating machine fault diagnosis.
